# Patient Navigation in Cancer Treatment: A Systematic Review

**DOI:** 10.1007/s11912-024-01514-9

**Published:** 2024-04-06

**Authors:** Matthew Chen, Victoria S. Wu, Derek Falk, Chesley Cheatham, Jennifer Cullen, Richard Hoehn

**Affiliations:** 1 Case Western Reserve University School of Medicine, Cleveland, OH USA; 2 Division of Surgical Oncology, Department of Surgery, University Hospitals Cleveland Medical Center, Cleveland, OH USA

**Keywords:** Patient navigation, Cancer, Treatment initiation, Treatment adherence, Quality indicators, Patient satisfaction

## Abstract

**Purpose of Review:**

Patient navigation promotes access to timely treatment of chronic diseases by eliminating barriers to care. Patient navigation programs have been well-established in improving screening rates and diagnostic resolution. This systematic review aimed to characterize the multifaceted role of patient navigators within the realm of cancer treatment.

**Recent Findings:**

A comprehensive electronic literature review of PubMed and Embase databases was conducted to identify relevant studies investigating the role of patient navigators in cancer treatment from August 1, 2009 to March 27, 2023.

**Summary:**

Fifty-nine articles were included in this review. Amongst studies focused on cancer treatment initiation, 70% found a significant improvement in treatment initiation amongst patients who were enrolled in patient navigation programs, 71% of studies focused on treatment adherence demonstrated significant improvements in treatment adherence, 87% of studies investigating patient satisfaction showed significant benefits, and 81% of studies reported a positive impact of patient navigators on quality care indicators. Three palliative care studies found beneficial effects of patient navigation. Thirty-seven studies investigated disadvantaged populations, with 76% of them concluded that patient navigators made a positive impact during treatment. This systematic review provides compelling evidence supporting the value of patient navigation programs in cancer treatment. The findings suggest that patient navigation plays a crucial role in improving access to care and optimizing treatment outcomes, especially for disadvantaged cancer patients. Incorporating patient navigation into standard oncology practice can reduce disparities and improve the overall quality of cancer care.

## Introduction

The intricate nature of cancer care can pose significant challenges for patients, leading to gaps in understanding, delays in treatment, and disparities in healthcare access. Patient navigation is a community-based intervention designed to increase access to care and eliminate barriers patients may face [1]. Beginning in the 1990s, patient navigation programs emerged as a promising approach to support patients through the cancer care continuum by providing personalized assistance and guidance to improve patient outcomes and reduce racial healthcare disparities [1]. Since then, these programs have emerged as a critical strategy aimed at addressing challenges and optimizing the patient journey through the complexities of cancer diagnosis, treatment, and survivorship. The roles of patient navigators are constantly evolving and expanding, with the Center for Disease Control and American Cancer Society dedicating more broader definitions regarding their use in healthcare [2, 3].

**Fig. 1:**
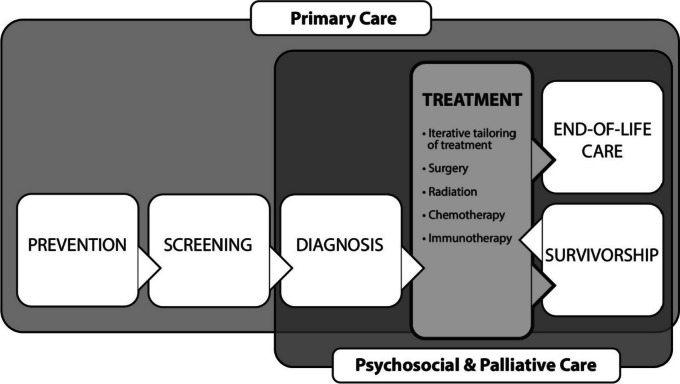
Cancer Care Continuum

The cancer care continuum is a comprehensive framework that outlines the various stages and elements involved in care for individuals affected by cancer [4]. Specifically, the “treatment” phase of the continuum is the time period between diagnosis and survivorship which includes curative-intent therapies, disease-control therapies, and symptom management [5]. Types of cancer treatment may include surgery, chemotherapy, radiation therapy, hormonal therapy, targeted therapy, and immunotherapy (Fig. 1). This continuum is designed to ensure a coordinated approach to managing cancer throughout a patient’s journey. The benefits of patient navigation have been thoroughly documented in phases of screening and diagnostic resolution [6]. However, there is a paucity of literature evaluating the efficacy of patient navigation on cancer treatment as a whole [7–12]. We conducted a systematic review to characterize the role of patient navigation in cancer treatment, focusing on treatment initiation, treatment adherence, quality indicators, palliative care, and patient satisfaction. The findings of this review could have important implications for cancer care providers, policymakers, and patients, for future implementation of navigation programs that improve cancer care delivery and patient outcomes.

## Methods

### Data Sources

A comprehensive electronic literature review of PubMed and Embase databases was conducted to identify relevant studies investigating the role of patient navigators in cancer treatment. Search terms were devised in coordination with a librarian to ensure pertinent literature, balance between specificity and inclusivity, and maintain a strong focus on patient navigation and cancer treatment. The following MeSH terms were used for PubMed: (("Neoplasms"[Mesh]) OR (cancer patient*[tiab])) AND (("Patient Navigation"[Mesh]) OR (patient navigat*[tiab])). The search term used for Embase was as follows: ('patient'/exp OR patient) AND ('navigator'/exp OR navigator) AND cancer AND [01–08-2009]/sd NOT [24–03-2023]/sd AND [2009–2023]/py.

We also summarized ongoing clinical trials for patient navigation in cancer treatment through the National Cancer Institute’s (NCI) clinicaltrials.gov. This search was filtered with the following: condition—cancer, terms—navigation, study status—not yet recruiting, recruiting, and enrolling by invitation. The search was conducted on March 24, 2023.

### Inclusion and Exclusion Criteria

Studies were selected for inclusion in this systematic review if they were published between the dates of August 1, 2009 to March 24, 2023. Articles included all cancer patients that received cancer treatment and had a patient navigation intervention group studied. Empirical research, randomized controlled trials, cohort studies, case–control studies, and observational studies were included. Studies also had to be published in English. Studies were excluded if they were published before 8/1/2009 or after 3/24/2023. Studies that did not implement patient navigation or were not directly related to the role of patient navigators in cancer treatment were excluded. Non-empirical studies, including reviews, commentaries, or editorials were excluded. Case studies presenting individual patient cases without broader empirical analysis were also excluded. Clinical trials were included if they focused on patient navigation during the cancer “treatment” phase defined by the Cancer Care Continuum [5].

### Study Selection

After removing duplicates with Excel string matching across titles, the titles and abstracts of the remaining records were screened independently by two reviewers (M.C. and V.W.) for relevance to the research question and adherence to the inclusion and exclusion criteria. Full-text articles were retrieved for records that met the initial screening criteria. The full-text articles were then assessed by the reviewers (M.C. and V.W.) for final inclusion in the systematic review.

### Quality Assessment

The methodological quality of the included studies was assessed using the 2018 version of the Mixed Methods Appraisal Tool (MMAT) [13]. The MMAT includes specific criteria for assessing the quality of various study designs, such as randomized controlled trials, cohort studies, case–control studies, and observational studies. This quality assessment aimed to evaluate the validity and reliability of the included studies and inform the interpretation of their findings.

### Data Synthesis

A narrative synthesis approach was used to summarize and analyze the findings of the included studies. Themes related to the impact of patient navigators on treatment initiation, treatment adherence, patient satisfaction, quality indicators, and palliative care were identified and discussed. Secondary themes included the country the study was performed in, the types of cancer patients had, and whether the study had a focused on a disadvantaged population such as racial minorities, socioeconomically challenged, and underinsured. The synthesis aimed to provide a comprehensive overview of the role of patient navigators in cancer treatment across different contexts and populations.

## Results

### Study Selection

**Fig. 2:**
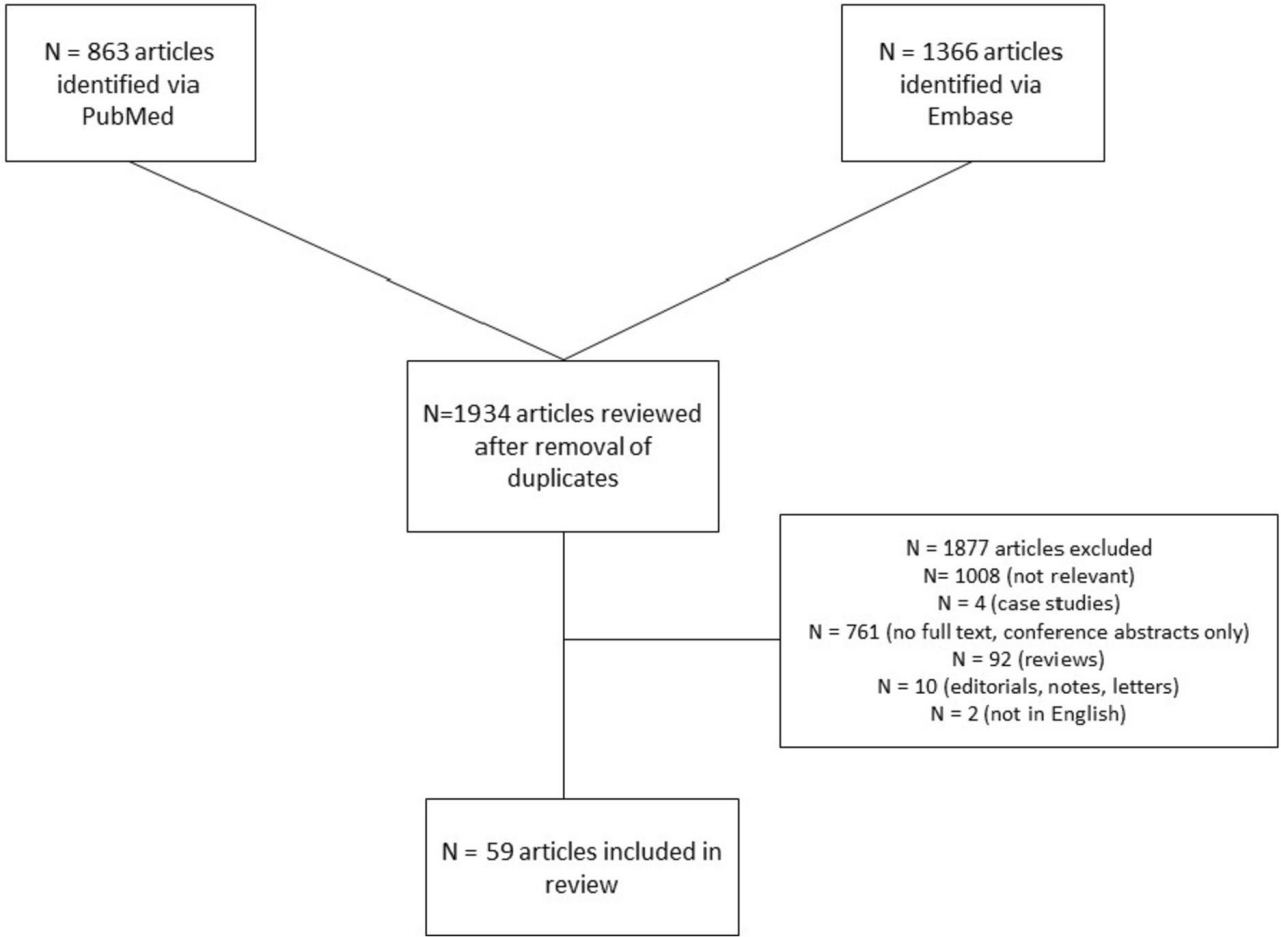
Articles included in the patient navigation and cancer treatment review

A total of 2229 articles were identified through the PubMed and Embase databases. Duplicates were removed and 1934 articles remained (Fig. 2). Of the remaining 1934 articles, 1876 were excluded based on inapplicable results or not meeting inclusion criteria. 1008 were not relevant to patient navigation during cancer treatment, 761 did not have a full text or were conference abstracts only, 92 were reviews, 10 were editorials, notes, or cover letters, 4 were limited case studies, and 2 were not written in English. Fifty-nine total articles were included in this review, with thirty-seven of them investigating disadvantaged groups.

### Treatment Initiation

Twenty-three articles focused on the impact of patient navigators on the time between diagnosis and treatment initiation. Of these, 70% (*n* = 16) reported a significant reduction in time to treatment with patients that received navigation. These positive outcomes were observed across various cancer types, including eight breast, three lung, one head and neck, one hematologic, one pancreatic, one gastrointestinal, and one multiple cancer studies [14–29]. Eight breast and one gastrointestinal cancer study focused on disadvantaged populations [14–21, 25]. The remaining studies either showed no significant difference or increased time to treatment between patient navigation groups and control groups [30–36]. A comprehensive overview of these findings is presented in Table 1.

**Table 1:** Treatment initiation

Study	Cancer(s) evaluated	Country of study	Age group/patient cohort	Disadvantaged population study (Y/N)	Outcomes studied	Key findings
Koh et al. (2011) [30]	Breast	USA	21 and older in a tertiary care facility	N	Treatment initiation	Time interval between diagnostic biopsy to initiation of cancer treatment was reduced from an average of 30 days (SD = 11.79; 95% CI 26.8, 33.2) to 26.2 days (SD = 9.15; 95% CI 22.9, 29.4) but was not statistically significant (*t* = 1.606, *p* = 0.112, Cohen’s *d* = 0.366)
Haideri and Moormeier (2011) [14]	Breast	USA	Truman Medical Center in Kansas City, Missouri, a safety net hospital for Kansas City	Y	Treatment initiation	The median time to first treatment was decreased by 9 days in the navigation group
Dudley et al. (2012) [15]	Breast	USA	Mentally competent patients 18 years of age from San Antonio, Texas, which has significant underserved populations typically associated with cancer health disparities: large Hispanic population, high illiteracy rate, low socioeconomics, and a large number of uninsured	Y	Treatment initiation	More navigated Hispanic women began treatment within 60 days of diagnosis (80% vs 56.3%, *p* < 0.01)
Mireles-Aguilar et al. (2018) [16]	Breast	Mexico	Patients with abnormal breast findings or imaging studies and guiding women in Nuevo Leon, Mexico,	Y	Treatment initiation	The median time from alert activation to treatment initiation was 33 days, and from first medical evaluation to treatment initiation was 28 days
Ramirez et al. (2014) [17]	Breast	USA	Latinas with breast cancer in community health based clinics	Y	Time from diagnosis to treatment initiation within 30 or 60 days	A higher percentage of navigated subjects initiated treatment within 30 days (66.7% versus 56.7%, *p* = 0.045) and 60 days (97.8% versus 78.4%, *p* = 0.021) following their cancer diagnosis compared to the control group
Rohsig et al. (2019) [18]	Breast	Brazil	Brazilian women in Hospital Moinhos de Vento (HMV), a private, nonprofit, 434-bed general hospital in Porto Alegre, Brazil	Y	Treatment initiation	In 2014, the mean time to treatment was 20.25 days. The maximum diagnosis-to-treatment mean time was 24.25 days in 2015, and the minimum time was 18.18 days in 2016 (the year in which the ONN program was set up)
Tamez-Salazer et al. (2020) [19]	Breast	Mexico	Women with breast symptoms or abnormal imaging studies in Nuevo Leon, Mexico. The program was created by a nongovernmental organization (NGO) focused on overcoming the challenges that impede early BC detection and improving the quality of life of vulnerable populations	Y	Treatment initiation	The median time from initial patient contact to treatment initiation was 39 days. A recent local analysis of mammography interpretation delays within a main referral public hospital found that the median time from date of imaging study to return of results alone was 39 days
Perez-Bustos et al. (2021) [20]	Breast	Colombia	Patients in a secondary healthcare provider Hospital in Cali (Colombia) by the Fundación para la Prevención y Tratamiento del Cáncer (FPTC) and Partners for Cancer Care and Prevention (PFCCAP), two sister organizations from Cali, Colombia, South America, and Baltimore, Maryland, USA, respectively	Y	Treatment initiation	Navigation decreased the interval from oncologist appointment to first chemo session or surgery from 87 to 15 days Navigation also decreased from the first chemotherapy cycle to the second chemotherapy cycle decreased from 57 to 24 days
Vieira et al. (2023) [21]	Breast	Brazil	Brazilian women in Hospital das Clínicas/Universidade Federal de Minas Gerais	Y	Treatment initiation Treatment completion	Patient navigation decreased the median time from the biopsy result to the beginning of radiation therapy from 108 to 74 days (*p* < 0.001) Also reduced the time between the referral to the end of the radiation therapy (98 to 78 days, *p* < 0.003)
Battaglia et al. (2022) [31]	Breast and lung	USA	Racially/ethnically diverse, low-income patients with cancer at the largest safety-net medical center in New England from February 2013 through August 2017	Y	Treatment initiation	Navigation enhanced by legal support did not result in more timely care over a 6-month period No significant effect of enhanced navigation was observed on the receipt of timely treatment among participants with breast cancer (odds ratio, 0.88; 95% CI, 0.17–4.52) or lung cancer (odds ratio, 4.00; 95% CI, 0.35–45.4)
Wagner et al. (2014) [32]	Breast, colorectal, lung cancer	USA	The trial was conducted in the Seattle and Bellevue service areas of Group Health (GH), an integrated, nonprofit delivery system serving 640,000 enrollees in Washington and Northern Idaho	Y	Treatment initiation	No significant difference was found between groups in the number of days between diagnosis and first oncology visit, and onset of treatment Control patients received their first surgery significantly earlier than NN patients (24 days v 30 days after diagnosis, respectively)
Freund et al. (2014) [33]	Breast, cervical, colorectal, prostate	USA	The majority of the sites were community health centers, in addition to several outpatient practice settings within and outside of safety-net hospitals. Most sites cared for primarily patients who were low income, uninsured or publically insured, and from racial and ethnic minority populations	Y	Treatment initiation	There was no benefit during the first 90 days of care, but a benefit of navigation was seen from 91 to 365 days for treatment initiation (aHR = 1.43; 95% CI = 1.10 to 1.86; *P* < 0.007) The navigated arm had a smaller proportion of participants who had initiated treatment at both 60 days (57% vs 62%) and 90 days (73% vs 75%) compared with the control arm. The findings were reversed at 365 days, and navigated participants had a higher proportion (89%) who had initiated treatment compared with the control participants (87%)
Kunos et al. (2015) [22]	Lung	USA	Summa Cancer Institute (Akron, OH)	N	Treatment initiation	During December 2009-September 2013, the time between the suspicion of cancer on chest X-ray to treatment was 64 days. During October 2013–March 2014, the nurse navigator significantly reduced that timespan to 45 days (*P* < 0.001)
Zibrik et al. (2016) [23]	Lung	Canada	BC Cancer Agency, Vancouver	N	Treatment initiation	Referral to systemic treatment was significantly reduced from 48 to 38 days (*P* = 0.016) Comparison of molecular testing showed time between referral and the epidermal growth factor (EGFR) result was significantly reduced from 34 days in 2011 to 20 days in 2014 (*P* < 0.001)
Hunnibell et al. (2012) [24]	Lung	USA	Patients diagnosed with NSCLC in The Connecticut Veterans Affairs Healthcare System	N	Treatment initiation	Baseline data reviewed from fiscal year 2003 showed an average of 117 days from suspicion to treatment. By 2007, the mean number of days from suspicion to treatment was 64.5. In 2010, the number had been reduced by almost two weeks to 52.4 days
Muñoz et al. (2018) [25]	GI	USA	Fresno County in California and addressed the diverse population of patients with GI cancer within the Community Medical Centers Healthcare Network, which includes the Community Regional Medical Center and the Clovis Community Medical Center	Y	Treatment initiation	Patients with a nurse navigator had a shorter time between diagnosis and treatment commencement (*p* < 0.001) The average time spent between initial diagnosis and the start of treatment was 15.15 days, compared to 42.93 days for patients not part of the multidisciplinary cancer care model
Dockery et al. (2018) [34]	Cervical	USA	The AI navigation program at the Stephenson Cancer Center, a tertiary care center	N	Treatment initiation	Median time to initiation of treatment was not different between navigation and control groups,, 30.5 days vs. 27.5 days (*p* = 0.18)
Gordils-Perez et al. (2017) [26]	Gynecologic and hematologic	USA	Women at a National Cancer Institute–designated comprehensive cancer center, Rutgers Cancer Institute of New Jersey in New Brunswick	N	Treatment initiation	A decrease of seven days from first oncologist consultation to start of treatment was observed between the historic (— X = 47.8, SD = 34.2) and post implementation (— X = 40.7, SD = 22.3) gynecology groups, but it was not statistically significant (*p* = 0.29) There was a statistically significant decrease to the first therapy from historic (— X = 27.1, SD = 28.5) to post implementation (— X = 16.0, SD = 9.7, t[88.94] = 3.21, *p* = 0.002) in the hematology population
Enomoto et al. (2019) [27]	Pancreatic	USA	Wake Forest Baptist Medical Center (WFBMC) is an NCI-designated, tertiary referral comprehensive cancer center	N	Treatment initiation	Days from first contact to treatment dropped from 46 to 26 days after starting Oncology Navigator program (*p* = 0.005)
Serrell et al. (2019) [35]	Prostate	USA	Men diagnosed with localized prostate cancer between 2009 and 2015 from the MaineHealth multi-specialty tumor registry, a non-profit, integrated, rural health system comprising 12 hospitals and healthcare networks including the Maine Medical Center Cancer Institute (MMCCI)	Y	Treatment initiation	Navigation was significantly associated with longer time to treatment (OR 1.65, 95% CI 1.12–2.45)
Ohlstein et al. (2015) [28]	Head and neck	USA	Tulane University School of Medicine Head and Neck Clinic between 2011 and 2014	N	Treatment initiation	An aspirational goal of treatment recommendation within 2 weeks of presentation was achieved for 47/93 patients established with a navigator. 83/93 received treatment recommendations within 1 SD and 89/93 within 2 SD of mean interval
Burhansstipanov et al. (2014) [29]	All	USA	Northern and Southern Plains American Indians	N	Treatment initiation	Most patients began receiving care within 1 month of a positive cancer biopsy. This time frame is a significantly shorter interval for treatment initiation reported elsewhere for American Indian cancer patients
Vilchis et al. (2019) [36]	All	USA	This program provided and evaluated services to cancer patients and their families in three counties in southwest (SW) New Mexico: Grant, Luna, and Hidalgo counties	Y	Treatment initiation	Mean time from cancer diagnosis to treatment initiation among 41 study patients was 59.6 days across the three counties. Mean time from non-intervention comparison data was 47.1 days

### Treatment Adherence

Seventeen articles delved into the impact of patient navigators in treatment adherence, measuring the extent to which patients adhered to treatment plans and attended appointments throughout their cancer journey. A total of 71% (*n* = 12) demonstrated a significant improvement of treatment adherence with navigation compared to the control group [37–48]. This was evident across multiple types of cancer, including five breast, three cervical, one osteosarcoma, one lymphoma, and two multiple cancer studies. Eight of these twelve studies focused on vulnerable populations, distributed amongst three breast, three cervical, one lymphoma, and one multiple cancer studies [37, 39, 41–44, 47, 48]. Five articles discovered no difference in treatment adherence between patients that received patient navigation and those who did not [25, 34, 49–51]. A comprehensive overview of these findings is presented in Table 2.

**Table 2:** Treatment adherence

Study	Cancer(s) evaluated	Country of study	Age group/patient cohort	Disadvantaged population study (Y/N)	Outcomes studied	Key findings
Ell et al. (2009) [49]	Breast and gynecological	USA	Patients in an urban public safety net medical center if they had a primary diagnosis of breast (stage 0–III) or gynecologic FIGO 0-4B cancer	Y	Treatment adherence	Overall adherence rates were (87–94%) and there were no significant differences between the navigated and usual care groups
Fouad et al. (2010) [37]	Breast	USA	Low income women, primarily African Americans, in 4 Alabama Counties (2 urban, 2 rural)	Y	Treatment adherence	Out of 1384 scheduled appointments, PNs successfully helped patients keep 1286 appointments, leading to an adherence rate of 93%
Fiscella et al. (2012) [50]	Breast and colorectal	USA	Rochester participants were primarily recruited from participating oncology practices (*n* = 13), both hospital and community-based. In Denver, participants were recruited from a single oncology practice within the Denver Health System, an integrated public safety net that includes a hospital and multiple health center sites	Y	Time to treatment completion	A total of 287 participants received chemotherapy or radiation therapy. All patients completed their treatment The median time to complete treatment (57 days for intervention and 63 days for control) was not statistically significantly different between the groups (*p* > 0.05) There were no statistically significant differences when results were stratified by cancer type, stage, or participant characteristics
Bickell et. al (2014) [51]	Breast	USA	Women with breast cancer from eight inner-city hospitals: four municipal and four tertiary referral centers	Y	Treatment adherence rates	High rates of intervention (INT) and usual care (UC) patients received treatment: 87% INT versus 91% UC women who underwent lumpectomy received radiotherapy (*P* = 0.39); 93% INT versus 86% UC women with estrogen receptor (ER) –negative tumors ≥ 1 cm received chemotherapy (*P* = 0.42); 92% INT versus 93% UC women with ER-positive tumors ≥ 1 cm received hormonal therapy (*P* = 0.80)
Ko et al. (2014) [38]	Breast	USA	Women with breast cancer who participated in the national Patient Navigation Research Program	N	Treatment adherence	Navigated participants eligible for antiestrogen therapy were more likely than non-navigated controls to receive antiestrogen therapy (OR, 1.73; 95% CI, 1.19 to 2.53; *P* = 0.004) Navigated participants who were eligible for radiation therapy were no more likely than controls to receive radiation (OR, 1.42; 95% CI, 0.80 to 2.54; *P* = 0.22)
Petereit et al. (2016) [39]	Breast	USA	Rapid City Regional Hospital (RCRH), a community based hospital which serves as a regional tertiary hospital providing health care services to three of the largest Indian reservations in the U.S. where an estimated 70,000 AIs reside. Additionally, according to the Index of Medical Underservice, western South Dakota, where our center is located, is designated medically underserved	Y	Treatment adherence to breast conservation therapy	Breast cancer patient navigation increased breast conservation rates (56% in navigated, 37% in non-navigated) Patients demonstrated a consistent and significant annual increase in treatment with BC versus a mastectomy (+ 2.9%/ year, *p*-trend < 0.001)
Benn et al. (2020) [40]	Breast	South Africa	Netcare Breast Care Centre of Excellence (BCCE), a single unit in Johannesburg South Africa that has been operational and running as a multidisciplinary breast care cenetr since 2000. The unit sees approximately 450 newly diagnosed breast cancer patients a year	N	Treatment adherence	80% of the code red patients, eventually agreed to recommended treatment. All Code Yellow patients completed their chemotherapy regimens during the course of the study, as did all Code Green patients
Čačala et al. (2021) [41]	Breast	South Africa	Conducted at Grey’s Hospital, Pietermaritzburg, KwaZulu-Natal, South Africa, which has a population of approximately 3.5 million, largely rural and low income	Y	Treatment adherence	In the non-navigated group 1, 40.2% (113/281) did not complete their primary chemotherapy course, compared with 13.5% (21/154) in the navigated group, *p* < 0.00001 In the control group, therapeutic breast surgery was performed on 103/181 (56.9%) patients initially eligible (stage 3 disease) compared with 66/81 (81.5%) of navigated patients, *p* < 0.0001
Luckett et al. (2015) [42]	Cervical	USA	Tertiary care referral colposcopy center	Y	No show rates	African American, Hispanic, and publicly insured women tended to miss appointments more frequently than did white and privately insured women (*p* < 0.0001) No-show rates declined from 49.7 to 29.5% after implementation of the patient navigator program (*p* < 0.0001) 45% of patient no-shows were anticipated or a result of patient misunderstanding and could be mediated with targeted education by the patient navigator
Dockery et al. (2018) [34]	Cervical	USA	The AI navigation program at the Stephenson Cancer Center, a tertiary care center	N	Treatment adherence	Of 55 patients identified, 34 received navigation and 21 did not. There was no difference in completion of prescribed therapy between groups (92% navigated vs 100% pre-navigation)
Dessources et al. (2020) [43]	Cervical	USA	Patients treated at Olive View–UCLA Medical Center (OVMC)—1 of the 4 large, safety-net hospitals of the Los Angeles County Department of Health Services	Y	Treatment adherence	After navigation implementation, the percentage of patients receiving ≥ 5 cycles of weekly cisplatin increased from 74 to 93% (*P* < 0.01) and rates of the initiation of brachytherapy during external beam radiotherapy increased from 49 to 78% (*P* < 0.01) The median treatment time was reduced from 67 days in the non-navigated patients to 55 days in the navigated patients (*P* < 0.01) 95% of navigated patients who completed pCRT did so within 63 days, compared with 52% of nonnavigated patients (*P* < 0.01)
Salcedo et al. (2021) [44]	Cervical	USA	The Rio Grande Valley (RGV) along the Texas–Mexico border has cervical cancer incidence and mortality rates approximately 25% higher than the rest of the state, and 55% higher compared with the rest of the US. more than 85% of the population is Hispanic or Latinx. In this region, 30% of the population live below the poverty line and approximately 40% have no health insurance	Y	Treatment adherence	A total of 2030 women (13.7%) were referred for colposcopy for abnormal results. A total of 453 LEEPs were performed during the project period. The number of women undergoing colposcopy and LEEP increased steadily over the course of the project period In 2018, the last year of intervention, an average of 73.5 women per month received colposcopy, a 179% increase compared with a baseline of 26.3 per month in 2013
Gaston et al. (2021) [45]	Osteosarcoma	Philippines	Philippine General Hospital from January 2016 to June 2019	N	Treatment abandonment rates	Treatment abandonment rates for the Pre-Patient Navigator group was significantly higher compared to those with a patient navigator (50 vs 6%, *p* = 0.0001)
Percac-Lima et al. (2015) [46]	GI, head and neck, and hematologic	USA	Cancer patients at ambulatory clinics of the Massachusetts General Hospital Cancer Center (MGHCC)	N	No show rates	The no show rate for the GI clinic was 12.5% in the control arm versus 9.6% in the intervention arm (*P* = 0.001). In the hematologic malignancy clinic, the no show rate was 11.9% in the control group versus 4.3% in the intervention group (*P* = 0.006). The head and neck oncology clinic had an NSR of 14.7% in the control versus 9.2% in the intervention group (*P* = 0.193)
Muñoz et al. (2018) [25]	GI	USA	Fresno County in California and addressed the diverse population of patients with GI cancer within the Community Medical Centers Healthcare Network, including the Community Regional Medical Center and the Clovis Community Medical center	N	No show rates	Statistical analysis revealed no difference in missed appointment rates between the two groups (*p* = 0.7)
Koffi et al. (2019) [47]	Lymphoma	Ivory Coast	Clinical Hematology Department of Abidjan University Medical Center (Ivory Coast)	Y	Treatment refusal rates	The navigated group displayed significantly lower rates of refusal and abandonment, compared to controls (*p* = 0.046)
Guadagnolo et al. (2011) [48]	All	USA	American Indian cancer patients presented to Rapid City Regional Hospital (RCRH) Cancer Care Institute (CCI) in Rapid City, South Dakota	Y	Treatment interruptions	Navigated patients had fewer days of treatment interruption (mean, 1.7 days; 95% CI, 1.1–2.2 days) than historical controls who did not receive PN services (mean, 4.9 days; 95% CI, 2.9–6.9 days)

### Patient Satisfaction

Fifteen articles assessed patient satisfaction or quality of life, which explored the contentment of patients with their care during the treatment process. Thirteen out of the fifteen studies (87%) demonstrated high rates of satisfaction with their navigator [18, 26, 30, 36, 50, 52–59]. Many of these studies encompassed several different cancers, ranging from breast, cervical, colorectal, prostate, head and neck, melanoma, gynecologic, and hematologic malignancies. Five of these studies, investigating all cancers and breast cancer, were done on disadvantaged groups [18, 36, 50, 58]. Two articles found no significant difference in satisfaction with patient navigation during treatment [60, 61]. A comprehensive overview of these findings can be found in Table 3.

**Table 3:** Patient satisfaction

Study	Cancer(s) evaluated	Country of study	Age group/patient cohort	Disadvantaged population study (Y/N)	Outcomes studied	Key findings
Koh et al. (2011) [30]	Breast	USA	Tertiary care facility	N	Patient satisfaction	32 women were approached to participate in the patient satisfaction survey after initiation of their cancer treatment, with 30 (94%) responding positively
Rohsig et al. (2019) [18]	Breast	Brazil	Brazilian women with breast cancer in Hospital Moinhos de Vento (HMV), a private, nonprofit, 434- bed general hospital in Porto Alegre, Brazil	Y	Patient satisfaction	153 patients responded to a patient satisfaction survey. 97% were satisfied or very satisfied with the care provided by the nurse navigator
Fiscella et al. (2012) [50]	Breast and colorectal	USA	Participants in a randomized controlled trial for PN from September 2006 to June 2010 at the two study sites. In Rochester, participants were primarily recruited from participating oncology practices (*n* = 13), both hospital and community-based. In Denver, participants were recruited from a single oncology practice within the Denver Health System, an integrated public safety net that includes a hospital and multiple health center sites	Y	Patient satisfaction	The median Patient Satisfaction with Cancer Care score was 81.7 with a standard error of 2.13 There was no significant difference in the proportion of patient navigation and control group patients who had a higher satisfaction score. However, we observed significant interactions between treatment group and language (*p* = 0.04), educational level (*p* = 0.007), and health insurance (*p* = 0.006) Being randomized to navigation was associated with significantly greater likelihood of higher satisfaction with cancer care among participants with lower English proficiency (OR 3.75; 95% CI 1.60–8.79), less than a high school education (OR 2.37; 95% CI 1.28–4.40), and no health insurance (OR 2.36; 95% CI 1.41–3.93)
Post et al. (2015) [60]	Breast, cervical, colorectal	USA	18 clinics in Central Ohio were randomized to either receive PN or a comparison condition	N	Patient satisfaction with cancer care, interpersonal relationship with navigator, and barriers to care	No significant difference was found between intervention and control groups in mean increase in patient satisfaction with cancer care (PSCC) from baseline to end-of-study. Although the difference was non-significant, participants in the intervention group had a higher mean increase in PSCC over time Intervention group participants’ satisfaction with their navigator was high (scores ranged from 9 to 45; mean = 40.19, SD = 5.91)
Wells et al. (2016) [61]	Breast, colorectal, cervical, and prostate	USA	Eight participating sites located in Boston, Chicago, Denver, Columbus, Ohio; Rochester, San Antonio, and Tampa approved this study. Sites of participant recruitment included federally qualified and hospital affiliated primary or outpatient specialty care clinics	N	Patient satisfaction	The PN group did not show significantly greater odds of having satisfaction with cancer-related care scores above the median when compared to the control group within 3 months of initiating cancer treatment (*p* > 0.05)
Gabitova and Burke (2014) [52]	Breast	USA	Northern California safety-net hospital Breast Clinic	Y	Patient satisfaction	More than 90% of the patients agreed that their navigator was friendly and respectful. 74% of patients felt that their navigator was sensitive
Jean-Pierre et al. (2013) [53]	Breast, Cervical, Colorectal, Prostate	USA	The PNRP enrolled adult participants (18 years and older) based on two primary criteria: (1) abnormal screening requiring diagnostic follow-up or (2) cancer diagnosis	N	Patient satisfaction	Navigation by better-rated navigators was associated with a greater likelihood of having higher patient satisfaction [odds ratio (OR), 1.38; 95% confidence interval (CI), 1.05–1.82] Similar findings between better-rated navigators and scores on the PSCC were found for participants with diagnosed cancer (OR, 3.06; 95% CI, 1.56–6.0) Patients navigated by better-rated navigators reported higher satisfaction with their cancer-related care
Berezowska (2019) [54]	Breast and melanoma	Netherlands	Netherlands Cancer Institute	N	Patient satisfaction	90% of patients who completed both the intervention and the questionnaire (*N* = 120, response rate 54%) perceived patient navigation as valuable, accessible, and reliable
Gordils-Perez et al. (2017) [62]	Gynecologic and hematologic	USA	Women at a National Cancer Institute–designated comprehensive cancer center, Rutgers Cancer Institute of New Jersey in New Brunswick	N	Patient satisfaction	Mean satisfaction survey scores for both groups were high regarding relationships with the navigator and care received. The PSN-1 survey revealed favorable responses
Fillion et al. (2009) [59]	Head and neck	USA	Patients with head and neck cancers followed for the first time at the oncology clinic of the university hospital for the reference period	N	Patient satisfaction	The study indicated an association between the presence of the navigator with continuity of care (higher satisfaction and shorter duration of hospitalization) and empowerment (defined by fewer cancer-related problems and better emotional quality of life)
Lee et al. (2011) [56]	All	South Korea	Patients who visited outpatient clinics of 2 branch hospitals of a university medical center in Korea	N	Patient satisfaction	Significant differences in satisfaction with care was observed between the 2 groups (*F* = 4.62, *P* = 0.001) Participants in the nurse navigator program (mean, 11.45 [SD, 3.69]) were more satisfied with the care compared with participants in the control group (mean, 14.95 [SD, 1.69]) (*F* = 11.85, *P* = 0.000)
Mir et al. (2022) [55]	All	France	Gustave Roussy Comprehensive Cancer Center and was open to patients with advanced or metastatic cancer started on approved oral chemotherapy and/or molecular-targeted therapy, not eligible for enrollment in another clinical trial	N	Patient satisfaction	Patient navigation improved the patient experience (Patient Assessment of Chronic Illness Care score, 2.94 versus 2.67, P = 0.01)
Berezowska et al. (2021) [57]	All	Netherlands	Patients newly diagnosed with ovarian, vulvar, endometrial, melanoma stage III/IV, lung, or renal cancer at the gynecology, lung, urology, and melanoma departments of the Netherlands Cancer Institute (NKI)	N	Patient satisfaction	The intervention group contained a higher percentage of patients who were (very) satisfied with the answers (8–47% of the intervention group was more satisfied than the control group), advice (7–26% of the intervention group was more satisfied than the control group), and empathy (1–22% of the intervention group was more satisfied than the control group) they received from healthcare professionals regarding supportive care issues
Vilchis et al. (2019) [36]	All	USA	This program provided and evaluated services to cancer patients and their families in three counties in southwest (SW) New Mexico: Grant, Luna, and Hidalgo counties	Y	Patient satisfaction	In the intervention group, on a 0–10 satisfaction scale (higher = more), patient mean scores ranged from 9.3 to 9.6
Guadagnolo et al. (2011) [58]	All	USA	American Indian patients presenting for cancer treatment and undergoing patient navigation at Rapid City Regional Hospital’s Cancer Care Institute in Rapid City, South Dakota	Y	Patient satisfaction	The mean scale score for satisfaction with health care was significantly higher after patient navigation compared with scores prior to navigation (*p* < 0.0001) with an increase of 0.41 (95% CI, 0.22–0.60) in the mean scale score

### Quality of Care

Eleven studies investigated the quality of patient navigation interventions by assessing adherence to various healthcare quality metrics such as hospitalizations, emergency department visits, financial assistance, or standard quality indicators tailored to different cancers. Nine (81%) of studies reported a positive impact of navigation on quality care indicators, which included four breast, one lymphoma, and four all-encompassing cancers studies [55, 56, 62–68]. Within these studies, two focused on breast cancer, one on lymphoma, and one involved all cancer types that targeted disadvantaged populations [56, 62, 64, 68]. Two studies found no improvement in cost of care or other healthcare assistance between navigated patients and the control [32, 69]. A comprehensive overview of these findings can be found in Table 4.

**Table 4:** Quality of care

Study	Cancer(s) evaluated	Country of study	Age group/patient cohort	Disadvantaged population study (y/n)	Outcomes studied	Key findings
Weber et al. (2012) [63]	Breast	USA	Women between the ages of 26 and 93 years with newly diagnosed breast cancer (invasive and noninvasive) undergoing treatment at East Carolina University Brody School of Medicine	N	Compliance to breast cancer care quality indicators (BCCQI)	There was improvement in the percentage of patients in compliance from pre and post implementation of a patient navigator program (range 2.5–27.0%) Overall, compliance with BCCQI improved from 74.1 to 95.5% (*p* < 0.0001)
Castaldi et al. (2017) [64]	Breast	USA	Patients at a public hospital in New York City over a 4-year period. This teaching-affiliated institution is a 450-bed acute care safety net public hospital serving one of the poorest boroughs of New York City and providing care to women who otherwise would have reduced access or none at all	Y	Compliance with three National Quality Forum measures	There was 100% compliance to National Quality Forum (NQF) measures in navigated care for all 3 therapies There was 57% compliance in chemotherapy, 68% compliance for hormonal therapy, and 85% compliance for radiation to NQF measures in usual care patients The navigated group had significantly higher rate of compliance to NQF measures in the chemotherapy and hormonal therapy, but not for radiation therapy
Wagner et al. (2014) [32]	Breast, colorectal, lung	USA	The trial was conducted in the Seattle and Bellevue service areas of Group Health (GH), an integrated, nonprofit delivery system serving 640,000 enrollees in Washington and Northern Idaho	Y	Cost of care	Cumulative costs were nearly identical in the 3 months before study enrollment At 12 months of follow-up, cumulative costs in the breast cancer and colorectal cancer NN groups tended to be slightly higher; however, cumulative costs in the NN arm of the lung cancer group were $6852 lower than the control group None of the differences in median cumulative costs between groups were statistically significant
Chen et al. (2010) [62]	Breast	USA	Public hospital of Los Angeles County	N	Breast cancer quality of care	Forty-nine patients were treated before the use of navigators and 51 after program implementation. Nine breast cancer quality indicators were used to evaluate quality of care Overall adherence to the quality indicators improved from 69 to 86 per cent with the use of patient navigators (*P* < 0.01). All nine indicators reached 75 per cent or greater adherence rates after implementation of the navigator program compared with only four before implementation
Raj et al. (2012) [65]	Breast	USA	Patients enrolled in the Massachusetts General Hospital (MGH) Avon Breast Care Program (MABCP), servicing disadvantaged minorities	N	Concordance to quality measures (QMs) of breast cancer	Patients who received navigation services received high-quality cancer care, as defined by concordance with ASCO/NCCN quality measures for hormonal therapy, chemotherapy, and radiation
Hu et al. (2021) [68]	Lymphoma	USA	Patients at a lymphoma clinic/transplant and cellular therapy program at the central location of the Levine Cancer Institute (LCI)	Y	Compare the outcomes of Whites and minorities in a lymphoma specialty clinic with a dedicated nurse navigator program	No significant differences between prognostic scores, frontline chemotherapy, or incidence in refractory disease between minority and white patients Minorities had more high-intensity encounters (42 vs 21%;* P* = 0.01) More minorities compared with Whites relied on nurse navigation for assistance with compliance concerns (18 vs 7%; *P* = 0.04), insurance questions (29 vs 8%; *P* = 0.002), financial concerns (37 vs 18%; *P* = 0.02), and transportation concerns (16 vs 2%; *P* = 0.004) High-intensity encounters were associated with significantly longer total times spent in comparison with low-intensity encounters (median, 135 vs 60 min; *P* < 0.001)
Yezefski et al. (2018) [67]	All	USA	Four hospitals in the USA participated in this study and received training by The NaVectis Group to implement a financial navigation program	N	Amount and type of assistance (free medication, new insurance enrollment, premium/co-pay assistance) patients received	Of 11,186 new patients with cancer seen across the 4 hospitals participating in the navigation program between 2012 and 2016, 3572 (32%) qualified for financial assistance They obtained $39 million in total financial assistance, averaging $3.5 million per year in the 11 years under observation Patients saved an average of $33,265 annually on medication, $12,256 through enrollment in insurance plans, $35,294 with premium assistance, and $3076 with co-pay assistance The 4 hospitals were able to avoid write-offs and save on charity care by an average of $2.1 million per year
Lee et al. (2011) [56]	All	South Korea	Patients who visited outpatient clinics of 2 branch hospitals of a university medical center in Korea	N	Length of hospital stay	The mean length of stay of participants in the experimental group (mean, 8.89 [SD, 3.63]) was significantly shorter than those in the control group (mean, 18.00 [SD, 11.89]) (*F* = 14.52, *P* = 0.000) Patients in the control group stayed in the hospital an average of 9.11 days longer than patients in the experimental group
Winget et al. (2020) [66]	All	USA	Cancer patients receiving multiple treatment modalities at the Stanford Cancer Institute	N	Emergency room and unplanned hospitalizations	Marginally lower incidence rate ratios (IRRs) for both ER visits (IRR, 1.17; 95% CI, 1.00 to 1.36) and unplanned hospitalizations (IRR, 1.18; 95% CI, 0.97 to 1.43) occurred in as-treated patients who used navigation help and who lived within 50 miles of Stanford Hospital compared with their matched controls
Mir et al. (2022) [55]	All	France	Patients at the Gustave Roussy Comprehensive Cancer Center and was open to patients with advanced or metastatic cancer started on approved oral chemotherapy and/or molecular-targeted therapy, not eligible for enrollment in another clinical trial	N	Adherence, toxicity, response and survival, quality of life, patient experience and economic estimation of the use of healthcare resources	Intervention reduced the days of hospitalization (2.82 versus 4.44 days, *P* = 0.02), and decreased treatment-related grade ≥ 3 toxicities (27.6% versus 36.9%, *P* = 0.02)
Lee et al. (2022) [69]	All	USA	Patients with cancer who received outpatient chemotherapy between January 1, 2018, and December 31, 2019, in a not-for-profit comprehensive community cancer center in an integrated healthcare system, Sharp HealthCare, in southern California	Y	The contribution of nurse navigators on healthcare utilization in the number of ED visits and hospital admissions of adults with cancer post–outpatient chemotherapy	The mean ranks for the number of ED visits (*U* = 4,053.5, *z* = –1.053, *p* = 0.292), average LOS at the ED (*U* = 4,449.5, *z* = 0.529, *p* = 0.597), number of hospital admissions (*U* = 15,472.5, *z* = 0.322, *p* = 0.747), and LOS at the hospital (*U* = 15,385, *z* = 0.135, *p* = 0.892); these were not significantly different for participants in terms of ONN involvement

### Palliative Care

There were three articles that investigated the role of patient navigation in palliative care for cancer patients during treatment. Two studies discovered an increase in compliance for advanced directive completion, in addition to increased supportive care efforts [70, 71]. The third study determined that navigators benefited family caregivers with decreased anxiety and improving their quality of life during treatment [72]. A comprehensive overview of these findings can be found in Table 5.

**Table 5:** Palliative care

Study	Cancer(s) evaluated	Country of study	Age group/patient cohort	Disadvantaged population study (Y/N)	Outcomes studied	Key findings
Fink et al. (2020) [71]	Advanced	USA	Hispanic patients, 18 years or older, with stage III/IV advanced cancer from 3 urban and 5 rural cancer center clinics across Colorado	Y	Palliative care outcomes	Navigated patients were more likely to have a documented AD compared with control group patients (73 of 112 [65.2%] vs 40 of 111 [36.0%], *P* < 0.001 Navigators also motivated patients to talk with their provider about pain needs with the intent to receive optimal pain management, and helped patients/family caregivers learn more about hospice
Soto-Perez et al. (2021) [70]	Advanced colon	Mexico	Patients with metastatic solid tumors from the oncology clinics at Instituto Nacional de Ciencias Médicas y Nutrición Salvador Zubirán (INCMNSZ), a public hospital in Mexico City	Y	The implementation of supportive care interventions and advanced directive completion	Supportive care interventions were provided to 74% of patients in the patient navigation arm versus 24% in usual care (difference 0.50, 95% confidence interval [CI] 0.34–0.62;* p* < 0.0001). In the patient navigation arm, 48% of eligible patients completed advance directives, compared with 0% in usual care (*p* < 0.0001).
Dionne Odom et al. (2022) [72]	Advanced stage cancer		Oncology outpatient clinic at a large tertiary academic medical center in the Southeastern United States that included African American/Black and rural-dwelling groups	Y	To assess ENABLE (Educate, Nurture, Advise, Before Life Ends) Cornerstone—a lay navigator-led, early palliative care telehealth intervention for African American/Black and/or rural-dwelling family caregivers of individuals with advanced cancer	Over 24 weeks, the mean ± SE Hospital Anxiety and Depression Scale score improved by 0.30 ± 1.44 points in the intervention group and worsened by 1.99 ± 1.39 points in the usual care group (difference, − 2.29; Cohen *d*, − 0.32). The mean between-group difference scores in caregiver quality of life was − 1.56 (usual care − intervention; *d*, − 0.07)

### Ongoing Clinical Trials

Table 6 lists ongoing clinical trials for patient navigation during cancer treatment. Eighteen trials were identified with our search.

**Table 6:** Clinical trials investigating the role of patient navigators in cancer treatment

Clinical trial name	Clinical trial identifier	Country	Cancer(s) evaluated	Intervention	Primary outcomes measured
Impact Of Nurse Navigation Program on Outcomes in Patients With GI Cancers (ACCESS) [73]	NCT04602611	USA	GI Cancers	Oncology Nurse Navigation	Acute Care Utilization Overall Survival
Patient Navigation to Improve Patient-Centered Cancer Care [74]	NCT03226405	USA	All cancers	Patient Navigation Program	Adherence with Cancer Treatment Time from Cancer Diagnosis to First Oncology Appointment
pCHIP: Prostate Cancer Health Impact Program [75]	NCT04293406	USA	Prostate cancer	Decision navigation intervention where patients will meet with navigator, prior to their specialist treatment consultation	Patient satisfaction and feedback
Improving Adherence to EHT Among Breast Cancer Patients [76]	NCT02850939	USA	Breast cancer	1) a culturally sensitive, personalized and easy to use smartphone app 2) support from a patient navigator	Change in adherence to endocrine hormonal therapy
Addressing Cancer-Related Financial Toxicity in Rural Oncology Care Settings [77]	NCT04931251	USA	All cancers	Financial Navigation	COST (Comprehensive Score for Financial Toxicity) measure
Navigation on Head and Neck Radiotherapy [78]	NCT04857749	Turkey	Head and neck cancer	Nursing Navigation	Quality of life measurements
Navigate—Improving Survival in Vulnerable Lung Cancer Patients [79]	NCT05053997	New Zealand	Lung cancer	Nurse Navigation	Overall Survival
Telehealth Based Synchronous Navigation to Improve Molecularly-Informed Care for Patients With Lung Cancer (TESTING) [80]	NCT05790460	USA	Lung cancer	Telehealth Nurse Navigation for early integration of concurrent molecular testing	Receipt of a molecularly-informed treatment recommendation for patients with metastatic NSq NSCLC at the time of the patient's initial oncology visit
Financial Navigation Program to Improve Understanding and Management of Financial Aspects of Cancer Care for Patients and Their Spouses (CREDIT) [81]	NCT04960787	USA	Hematopoietic and lymphoid cell neoplasm, metastatic solid neoplasm, recurrent solid neoplasm	Financial Navigation Program	Level of household financial hardship
Cancer Financial Experience (CAFE)[82]	NCT05018000	USA	All cancers	Financial Navigation	Financial Distress Health-related quality of life
Translating Research Into Practice (TRIP) [83]	NCT03514433	USA	Breast cancer	Patient Navigation	Time-to-treatment post-diagnosis
Multi-Site Trial of Navigation vs Treatment as Usual for Delays in Starting Adjuvant Therapy (ENDURE) [84]	NCT05793151	USA	Head and neck cancer	ENDURE: theoretically informed, navigation-based, multilevel intervention targeting barriers to timely, equitable guideline-adherent PORT	Initiation of post-operative radiation therapy
Rural Lung and Head and Neck Cancer Intervention [85]	NCT04916990	USA	Lung, head and neck cancer	Nurse navigators and masters levels counselors	Time to care
A Multilevel Intervention to Improve Timely Cancer Detection and Treatment Initiation (Potlako +) [86]	NCT04141449	USA	Multiple (cervical, breast, HNSCC, vulvar, anal)	Combined provider, patient, and health system intervention to expedite cancer diagnosis and care	Time to diagnosis Time to treatment Proportion of patients treated Curative incidence
Mobile Intervention to Improve Adherence of Oral Anti-cancer Medications Among Acute Myeloid Leukemia Patients, the txt4AML Study [87]	NCT05595135	USA	AML	Text Message-Based Navigation	Medication adherence
Assessing the Impact of a Financial Navigation Program for Patients With Multiple Myeloma [88]	NCT05448196	United States	Multiple myeloma	Coordinated Financial Navigation Program	Comprehensive Score for Financial Toxicity (COST)
Navigation vs Usual Care for Timely Adjuvant Therapy for Patients With Locally Advanced HNSCC (NDURE2) [89]	NCT04030130	United States	Head and neck cancer	Multi-level patient navigation	Time from surgery to start of postoperative radiation therapy
Assessment of Financial Difficulty in Participants With Chronic Lymphocytic Leukemia and Multiple Myeloma [90]	NCT03870633	USA	Chronic lymphocytic leukemia, multiple myeloma	Participants undergo medical chart abstraction within 1 week and complete telephone interview over 30–45 min within 8 weeks after registration	Proportion of patients reporting financial difficulties in the past 12 months

## Discussion

This systematic review examined the role of patient navigation during cancer treatment and demonstrated that patient navigation programs can improve patient outcomes and can reduce inequities in treatment based on non-medical factors. Patient navigation programs can decrease time to initiate treatment, increase patient adherence to treatment, and improve patient satisfaction and quality of care. Many of these studies focus on high-risk groups (i.e., minority or low-income populations), which further highlights the impact of these programs.

Thirty-seven of the fifty-nine articles (63%) focused on vulnerable healthcare populations. Within these thirty-seven studies, twenty-eight (76%) of them had positive conclusions regarding the role of patient navigators during cancer treatment. Eight of the sixteen studies that found significant improvement in treatment initiation for navigated patients were studies focused on disadvantaged patients [14–21, 25] Eight of the twelve studies that observed enhanced treatment adherence rates were done in disadvantaged populations as well [37, 39, 41–44, 47, 48]. Marginalized populations often struggle navigating the complex healthcare system and experience many barriers that lead to them not receiving the care that they need. With the help of patient navigators, they get to work closely with underserved patients and personalize a plan with their healthcare team to ensure that the treatment regimen is viable for the patient. These navigators can assist patients from different backgrounds throughout their cancer journey and help improve abandonment rates, treatment delays, and lead to better health outcomes.

Although the research is limited on the impact navigators have on palliative care, the three articles identified in this review consistently highlight favorable outcomes linked to the utilization of navigation services. The studies have demonstrated increased emotional and psychosocial support for both the patient and their family members. Patient navigators possess the capacity to assist patients in navigating the complex and challenging journey of managing a cancer diagnosis, relieving suffering, and providing support near the end of life through education, care coordination, and advance care planning. Given these promising effects but limited data, more research is necessary to understand the potential impact patient navigation programs hold in palliative care.

Our clinical trial search contained themes similar to our literature review. One trial utilized a culturally sensitive and personalized smartphone app, in addition to patient navigation, to assess change in adherence to endocrine hormone therapy amongst breast cancer patients [76]. The mobile app augments the patient navigation intervention by providing at-home educational content to patients in terms of potential side effects, management of symptoms, self-care skills etc. Another study utilizes text message navigation to provide patients with guidance while considering convenience for patients [87]. The American Cancer Society also recently launched their ACE CARES app which provides services for patients throughout their cancer journey [91]. Providing valuable information and navigation for cancer patients just at their fingertips could immensely improve their cancer journey. It could facilitate easier communication with their healthcare team and be more convenient overall for the patient. Digital literacy among users will need to be considered, and more research will be needed to fully understand its implementation and benefits for navigation.

It is notable that a significant proportion of the reviewed studies primarily focused on the impact of patient navigators in breast cancer care. While these studies provide valuable insights into the potential benefits of patient navigation, there exists a notable gap in the representation of other cancer types. This overrepresentation of breast cancer calls for increased attention to other cancer types to ensure a comprehensive understanding of the role of patient navigators across diverse oncological landscapes. The intricacies of treatment regimens, patient experiences, and healthcare disparities can vary substantially among different cancer populations. Diversifying the scope of investigation would not only provide a more nuanced understanding of the impact of patient navigators but also shed light on tailored strategies that could benefit patients facing less frequently studied cancers.

The recent decision by the Centers for Medicare and Medicaid Services (CMS) to reimburse patient navigation services for cancer patients is a significant step forward in enhancing the quality of care for individuals grappling with cancer [92]. Previously, navigation services were not billable, meaning that hospitals had to fund these services themselves. Now hospitals can use reimbursement codes for patient navigation services for cancer patients. This makes it more feasible for hospitals to allocate resources towards patient navigation. The decision not only recognizes the value of patient navigation in improving healthcare outcomes but also aligns with the broader healthcare trends of emphasizing patient-centered care and value-based reimbursement models. By reimbursing these services, CMS encourages healthcare providers to invest in comprehensive patient care and promotes a holistic approach to cancer treatment.

There are limitations to this review. Unpublished works and conference abstracts were not included, which may have created a potential for a lack of all-encompassing information. However, our search criteria was broad and allowed us to evaluate many publications. By limiting studies to English, there may be a lack of generalizability for international patient navigation programs. Studies that implemented patient navigation with cancer patients but not during their cancer treatment timeline were excluded. These studies may provide insight on the benefits of patient navigation, but this review was specifically focused on the duration of cancer treatment.

## Conclusion

In conclusion, this systematic review suggests compelling evidence supporting the role of patient navigators in cancer treatment. The studies in this review revealed how patient navigation can improve treatment initiation, adherence, quality of care, and patient satisfaction for many cancer patients, especially in disadvantaged populations. With upcoming policy changes improving the cost-effectiveness of these programs it is our hope that more widespread adoption may take place, and subsequently more patients will benefit from these valuable services.

## Data Availability

No datasets were generated or analyzed during the current study.
